# The role of SIRT1 in the development of gastrointestinal tumors

**DOI:** 10.3389/fcell.2025.1606530

**Published:** 2025-06-11

**Authors:** Yueming Zhang, Xiaokai Zhou, Jinghui Zhai, Jie Ma, Sixi Zhang

**Affiliations:** ^1^ Department of Clinical Pharmacy, The First Hospital of Jilin University, Changchun, Jilin, China; ^2^ Clinical Medical College, The First Hospital of Jilin University, Changchun, Jilin, China

**Keywords:** SIRT1, colorectal cancer, gastric cancer, tumor progression, chemoresistance

## Abstract

Gastrointestinal tumors, including esophageal cancer (EC), gastric cancer (GC) and colorectal cancer (CRC) and, pose significant global health challenges due to their high morbidity and mortality rates. SIRT1, an NAD^+^-dependent deacetylase, plays diverse roles in physiological processes and has been implicated in cancer development. This review examines the dual roles of SIRT1 in gastrointestinal tumors. In EC, SIRT1 consistently promotes tumor progression, with high SIRT1 expression associated with advanced TNM stage, poor prognosis, lymph node metastasis, and inferior overall survival. In GC, SIRT1 similarly promotes tumor progression via autophagy and chemoresistance, but studies also highlight its potential anti-cancer effects through ferroptosis regulation. In CRC, SIRT1 is often overexpressed and promotes tumor progression through mechanisms involving p53 inhibition, activation of the Wnt/β-catenin pathway, and regulation of Epithelial-Mesenchymal Transition (EMT). However, conflicting evidence suggests SIRT1 can also act as a tumor suppressor by inhibiting β-catenin and nuclear factor-κB (NF-κB) signaling. The dual nature of SIRT1 underscores the need for context-specific understanding of its function. Future research should focus on elucidating SIRT1’s mechanisms and developing personalized therapeutic strategies targeting SIRT1.

## 1 Introduction

According to the data compiled by the World Health Organization and the International Agency for Research on Cancer in 2022, cancer is indeed a significant cause of death globally, particularly among those under the age of 70 (Bray et al., 2021). Among them, colorectal cancer (CRC) accounted for 1,926,425 new cases, ranking third among the top ten most common cancers with second mortality. Gastric cancer (GC) had approximately 968,000 new cases globally, ranking fifth among all cancers. It also accounted for around 660,000 deaths, making it the fifth leading cause of cancer-related mortality (WHO, 2025). The high incidence and mortality rates for gastrointestinal tumors are largely attributed to factors such as chronic infection with *Helicobacter pylori*, dietary habits, and genetic predispositions. With the aggravation of population aging and the trend of cancer occurring at a younger age, the difficulty of prevention and treatment is self-evident. The burden of prevention and treatment for gastrointestinal tumors is becoming increasingly heavy. Therefore, the search for highly specific and sensitive biomarkers for early diagnosis of them and the implementation of precise targeted interventions with effective innovative drugs are extremely important.

Sirtuins (SIRT1-7) are NAD^+^-dependent deacetylases and mono-ADP-ribosyl transferases that are associated with extensive biochemical processes like inflammation, regulation of energy metabolism, DNA repair (Mich et al., 2007). Among them, SIRT1 is the widest studied sirtuin and is highly expressed in the liver, pancreas, heart, muscle, brain, and adipose tissue (Ceballos et al., 2023). It is primarily localized in the nucleus as an important regulatory enzyme (Kupis et al., 2016). In the energy metabolism of liver, SIRT1 activates the peroxisome proliferator-activated receptor (PPAR) γ coactivator 1α (PGC-1α) (Rodgers et al., 2005), nuclear receptor PPARα (Purushotham et al., 2009) to increase glucose generation and repress glycolytic enzymes (Hallows et al., 2012) to decrease glycolysis both by deacetylating. What’s more, several studies have demonstrated that SIRT1 play a significant role in the process of inflammation, researchers delete SIRT1 in *mouse* hepatocytes and find that the liver-specific SIRT1 knockout is easier suffer from hepatic steatosis and hepatic inflammation (Purushotham et al., 2009). For DNA repair, as a NAD^+^-dependent deacetylase, SIRT1 consistently localizes to the damage regions of DNA and deacetylates other proteins involved in DNA repair both *in vitro* and in cells (Fan and Luo, 2010). Interestingly, while SIRT1 is essential for maintaining normal physiological functions, it can also contribute to disease and discomfort under certain circumstances.

SIRT1 is often overexpressed in cancers occurring in the aforementioned tissues (Ceballos et al., 2023; Wauters et al., 2013). Notably, the relationship between CRC and SIRT1 has been extensively studied, with conflicting findings regarding its function (Menssen et al., 2012). For example, *Jang et al* analyzed 680 tissue samples from CRC patients at different stages and found that SIRT1 expression declines as the tumor progresses to a more malignant state (Jang et al., 2012). Additional studies have also suggested that SIRT1 exerts an inhibitory effect on CRC, with a focus on its role in tumorigenicity and metastasis (Sun et al., 2017; Harada et al., 2016; El-Kott et al., 2021), and its potential as a drug target (García-Martínez et al., 2023; Jung et al., 2015). Conversely, numerous studies have highlighted the positive effects of SIRT1 on CRC. These studies have explored various aspects, including the mechanisms of action of drugs used to treat CRC, such as 5-Fluorouracil, oxaliplatin (Ueno et al., 2013), fentanyl (Zhang et al., 2014), resveratrol (Buhrmann et al., 2017), as well as the pathogenesis of CRC through molecular pathways like p53/miR-101/KPNA3 (Wang et al., 2023), AKT/TM4SF1 (Wang R. et al., 2021), ATGL/ mTOR (Su et al., 2023) and so on. Similarly, this phenomenon is also observed in research on GC and SIRT1. There are also discussions regarding the molecules P53 (Sun et al., 2012), AKT (Fu et al., 2021) and miRNA (Li et al., 2021), as well as the drugs 5-FU (Zhao et al., 2015) and resveratrol (Yang et al., 2013a). Similar to CRC, SIRT1 exhibits a dual role in GC, with evidence supporting both its promotion and inhibition of cancer progression. Up to now, there remains no consensus regarding the role of SIRT1 in gastrointestinal tumors. The present study compiles the most recent findings on SIRT1 in the context of gastrointestinal tumors and provides a comprehensive summary, with the aim of facilitating future research endeavors.

## 2 Characteristics of SIRT1

**FIGURE 1:**
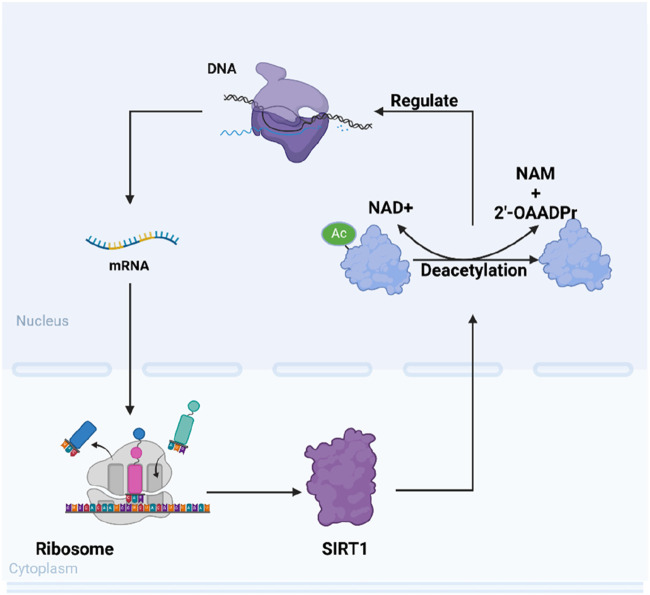
Schematic representation of SIRT1’s intracellular mechanisms of action. SIRT1 modulates various cellular functions such as DNA repair, gene expression regulation, and cellular metabolism through its NAD^+^-dependent deacetylation activity.

Mammals possess seven sirtuins, designated as SIRT1 to SIRT7: a conversed NAD^+^-dependent catalytic core domain that may act preferentially as a mono-ADP-ribosyl transferase and/or NAD^+^-dependent deacetylase. And both sides of the core domain connects additional N-terminal and/or C-terminal sequences of variable length (Frye, 2000). Specifically, the core domain of SIRT1 is solely responsible for deacetylation. Mammalian sirtuins also different in their sub-cellular localization. Even though SIRT1 has many vital functions in the cytoplasm, SIRT1 is mainly found in the nucleus (Michishita et al., 2005). The changing of intracellular ratio of NAD^+^/NAM will be caught by SIRT1 and affect its activity and substrate preference (Cantó et al., 2015). In this way, SIRT1 deacetylates different histones and other chromosomal proteins to transmit different signal of cellular metabolic status, ultimately altering gene expression (Vaquero, 2009). After that, its impact on human physiological functions is mainly manifested in the following aspects: promoting fat mobilization, stimulating the browning of white adipose tissue, controlling insulin secretion in the pancreas, sensing nutrient availability in the hypothalamus, regulating glucose and lipid metabolism in the liver, influencing obesity-induced inflammation in macrophages, and modulating the activity of the circadian clock in metabolic tissues (Li, 2013) (Figure 1).

## 3 SIRT1 and EC

SIRT1 consistently promotes the progression of esophageal cancer (EC). Numerous investigations have analyzed tissues and EC cell lines from EC patients, particularly those with esophageal squamous cell carcinoma (ESCC), revealing that high SIRT1 expression is associated with advanced TNM stage (Ha et al., 2018), poor prognosis (He et al., 2016), lymph node metastasis (Yan et al., 2020), inferior overall survival (Ma MC. et al., 2018), and other adverse factors. These findings collectively suggest that SIRT1 may function as an independent prognostic predictor in EC. Studies have demonstrated that the EMT process in EC cells can be inhibited by the application of SIRT1 inhibitors in both EC cell lines and mouse models (Qin et al., 2018). This inhibitory effect may be associated with SIRT1-mediated autophagy (Zhang et al., 2024). Additionally, several studies have identified key molecules that facilitate metastasis in esophageal cancer (EC), including lncRNA MNX1-AS140 (Chu et al., 2019) and the Cystathionine-β-synthase (CBS)/H_2_S (Liu et al., 2022) system. These molecules enhance the metastatic potential of EC by augmenting the activity of downstream SIRT1. Regarding chemoradiotherapy resistance, miR-34a is recognized as an upstream inhibitory molecule of SIRT1. Research has shown that treatment of *RECA109* cell lines with miR-34a can reverse their resistance to radiotherapy, an effect closely associated with the inhibition of SIRT1 (Ye et al., 2021). Futhermore, studies have demonstrated that genetic ablation of SIRT1 can enhance the sensitivity of cell lines to cisplatin (Yang et al., 2024; Morishita et al., 2024). Intriguingly, a recent study has correlated changes in the apparent diffusion coefficient (ADC) on MRI following chemoradiotherapy with SIRT1 levels, revealing that high SIRT1 expression is associated with diminished early efficacy of chemoradiotherapy (Chen et al., 2019).

## 4 SIRT1 and GC

The diverse biological functions of SIRT1 may explain the varied results observed in studies examining its relationship with GC. The number of studies suggesting that SIRT1 promotes GC is roughly equal to those indicating that SIRT1 inhibits GC. Therefore, we will elaborate on the role of SIRT1 in GC from both positive and negative perspectives.

### 4.1 Positive role of SIRT1 in GC

#### 4.1.1 Positive expression and influence of SIRT1 in GC

There are many studies have mentioned the high expression of SIRT1 in various GC types, such as gastric adenocarcinoma (Özcan et al., 2019), gastroesophageal junction cancer (Zhang LH. et al., 2013), gastric cardiac cancer (Feng et al., 2011), one of the studies reported that the expression level of SIRT1 in all stages of GC patient tissues (including precancerous lesions, early GC and advanced GC) was higher than that in non-cancerous gastric mucosa (Zhang S. et al., 2017). They found that the high expression of SIRT1 was statistically significantly correlated with the proliferation status of GC, advanced cancer stage (Jiang et al., 2016; Mohammadi Saravle et al., 2018), poor overall survival (OS) (Otsuka et al., 2023), recurrence free survival (Qiu et al., 2016), and increased number of metastatic lymph nodes (Cha et al., 2009; Noguchi et al., 2014). Notably, one study found that SIRT1 was associated with 3-year OS but not with 5-year OS (Jiang et al., 2016).

Besides, these studies also reported some molecules that interact with SIRT1. *Qiu et al* examined GC patients samples and found SIRT1 can deacetylate beclin-1 to mediate autophagy in GC cells, thereby participating in the progression of GC (Qiu et al., 2016). Similarly, the study of *Zhang et al* also reported comparable results. The expression level of SIRT1 was negatively correlated with the expression levels of E-cadherin and MutL Homolog 1 in patients’ tissues, which may also be related to the mechanism of SIRT1 (Zhang LH. et al., 2013). Other molecules positively correlated with SIRT1 include Ki-67^48^, STAT3(Signal Transducer and Activator of Transcription 3) and its activated form pSTAT3 (Zhang S. et al., 2017). Conversely, DBC1(Deleted in Breast Cancer 1) has been shown to indicate a better prognosis in patients when highly expressed (Cha et al., 2009; Noguchi et al., 2014).

These studies showed that SIRT1 is highly expressed in GC tissues and affects the progression of GC through multiple pathways, which is ultimately reflected in the prognosis of GC patients.

#### 4.1.2 SIRT1 promotes GC progression in multiple ways

P53, as an important target of SIRT1, is also essential in GC’s research. When *SGC7901 human GC* cells were transfected with VEGF siRNA, the expression of VEGF, SIRT1, survivin, and Bcl-2 was downregulated, while the expression of p53 and p21 was upregulated. This finding proved that the anti-cancer effect of VEGF siRNA was closely related to SIRT1/p53 pathway (Sun et al., 2012). Same as L-OHP, in addition to DNA, platinum drugs may also act on non-DNA molecules to promote their pro apoptotic effect and cytotoxicity. In gastric stomach cancer *(AGS)* cells, L-OHP can also act on tumor associated NADH oxidase, reduce the NAD^+^/NADH ratio, and reduce the activity of NAD^+^-dependent SIRT1, thereby enhancing the acetylation of p53 and apoptosis (Chen et al., 2017).

The regulation of SIRT1 based on NAD^+^/NADH ratio is also seen in other molecules, Ubiquitin specific protease 22 (USP22) is a deubiquitinase that is believed to be associated with various cancers. In *SCID mice*, USP22 regulates the transcription of genes associated with these processes by deubiquitinating H2A or H2B histones (Liu et al., 2019). USP22 can regulate SIRT1 through the c-myc/ Nicotinamide phosphoribosyltransferase (NAMPT) pathway, thereby affecting the Forkhead Box O1 (FOXO1) and Yes-associated protein (YAP) signaling pathways, which are respectively related to GC cells apoptosis and metastasis (Liu et al., 2019). In addition, another study reported the molecular mechanism of obesity promoting the development of GC, which also upregulated the expression of SIRT1 through the same pathway in diet-induced obese *mice* (Li et al., 2013).

In terms of the regulation of SIRT1, several studies have revealed that certain molecules can directly modify SIRT1 mRNA to modulate its expression. For example, IGF2BP2(Insulin-like Growth Factor 2 mRNA-Binding Protein 2) can directly act on the m6A modification site of SIRT1 mRNA, promoting the progression of GC through the IGF2BP2/SIRT1 axis both in cells line and *mice* xenograft model (Zhang Z. et al., 2022). MiRNAs also play a role in this process, miR-12129 can bind to the 3′-UTR of SIRT1 to inhibit the expression of SIRT1, thereby affecting the proliferation and cell cycle progression of GC cells (Zhang et al., 2020).

Another part of the research explored SIRT1 mediated chemoresistance in GC. Multidrug resistance (MDR) in GC has always been a challenge in clinical treatment, but its mechanism is still inconclusive. Notably, GC associated with diabetes often exhibits resistance to chemotherapy drugs, there is a study reported that the expression of NAMPT, SIRT1, p53, P-gp and Topo II α is higher in patients with both diabetes and GC, and the survival time is reduced. The proliferation rate of *SGC7901* cells increases at high glucose concentrations, while the inhibition rate of 5-FU on cells decreases over time. In mechanism, hyperglycemia may increase MDR by promoting the expression of NAMPT/SIRT1, which in turn promotes the expression of P-gp and reduces the expression of Topo-II α (Zhao et al., 2015). Further studies have identified molecules related to tumor MDR at the cellular level, such as downregulated miR-34a-5p and upregulated Activating Transcription Factor 4/(ATF4), both of which target SIRT1 (Deng et al., 2021; Zhu et al., 2012). These evidences further substantiate the intricate association between SIRT1 and the MDR phenotype in GC. In order to overcome the MDR of GC, new chemotherapy drugs targeting SIRT1 have also made progress. Jaridon 6, significantly inhibited the proliferation of drug-resistant GC cells and exhibited good cytotoxicity in cells and *nude mice* model. Jaridon 6 exerts its effects by inhibiting SIRT1 activity, further affecting the (Phosphatidylinositol 3-Kinase)PI3K/AKT signaling pathway, inducing autophagy, and promoting the death of drug-resistant cancer cells (Fu et al., 2021). Like CRC, the SIRT1 inhibitor Tenovin-6 also exerts anti-tumor effects in GC through the same mechanism (Hirai et al., 2014).

SIRT1 is also implicated in the metastasis of GC. Specifically, miR-204 overexpression reduces GC cell invasiveness and anti-apoptotic ability by targeting SIRT1, leading to decreased SIRT1 expression and subsequent modulation of EMT-related genes, such as increased E-cadherin and decreased vimentin levels. Conversely, miR-204 downregulation promotes GC cell invasion by activating the SIRT1- Liver Kinase B1(LKB1) signaling pathway (Zhang L. et al., 2013). A recent study found that USP14, which stabilizes SIRT1 through deubiquitination, is linked to poor prognosis and an immunosuppressive phenotype in GC patients. USP14-mediated SIRT1 stabilization promotes M2 macrophage polarization via SIRT1/PGC1-α-mediated lipid oxidation in *mouse* tumor models. While USP14 overexpression alone is not enough to polarize macrophages, inhibiting USP14 with IU1 can reshape the tumor microenvironment, underscoring SIRT1’s key role in the “cold tumor” phenotype (He et al., 2023).

**FIGURE 2:**
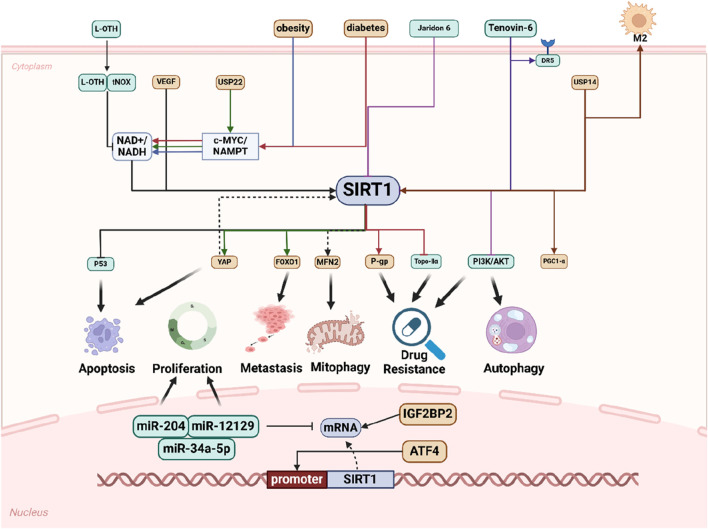
Schematic representation of SIRT1 promotes GC progression in multiple ways. Numerous extracellular factors influence intracellular molecular changes that affect processes such as cell apoptosis, proliferation, metastasis, mitophagy, drug resistance, and autophagy through distinct molecular pathways, with SIRT1 playing a pivotal role. Within the nucleus, SIRT1 regulates miRNAs and is itself regulated by other molecules to modulate cell phenotype.

The collective findings from the aforementioned studies offer substantial evidence that SIRT1 is intricately involved in various aspects of GC, including its initiation, proliferation, metastasis, autophagy, apoptosis, and the remodeling of the tumor microenvironment. As an oncogene, SIRT1 emerges as a pivotal player, exerting significant influence throughout the progression of GC (Figure 2).

### 4.2 Negative role of SIRT1 in GC

#### 4.2.1 Negative expression and influence of SIRT1 in GC

Despite numerous clinical studies indicating that SIRT1 is associated with poor prognosis in GC patients, conflicting results have also been reported. Based on the statistics of gene expression samples of 1065 GC patients in the database, it was finally found that as a prognostic marker, SIRT1 was associated with longer OS (Szász et al., 2016). Besides, some studies also reported that in SIRT1 positive GC tissues, the mRNA and protein levels of SIRT1 were lower than those in normal gastric tissues (Yang et al., 2013b; Li et al., 2016).

#### 4.2.2 SIRT1 inhibits GC progression in multiple ways

In addition to the conflicting findings on SIRT1 expression and prognosis in GC, a substantial body of research has been dedicated to elucidating the mechanisms by which SIRT1 exerts inhibitory effects on various processes in GC. Among them, research on miRNAs accounts for a considerable proportion. Dishevelled binding inhibitor of beta catenin3 antisense 1 (DACT3-AS1) can regulate the expression of SIRT1 by sponging mir-181a-5p. Similarly, circ-SIRT1 could also sponge miR-132-3p/miR-212-3p to upregulate the expression of SIRT1. High levels of SIRT1 have been associated with inhibited tumor proliferation in *mouse* model (Li et al., 2021). However, the inhibitory effect on GC proliferation diminishes due to decreased cellular levels of SIRT1, which may be caused by the upregulation of certain miRNAs that target SIRT1 mRNA. miR-132 play a cancer promoting role by directly acting on the 3′UTR of SIRT1 mRNA and inhibiting its expression (Zhang L. et al., 2017). miRNA-543 can also inhibit the expression of SIRT1, ultimately promoting EMT in GC (Li et al., 2016; Shi et al., 2019). Besides, SIRT1 has been reported to inhibit cyclin D1, thereby inducing G1 phase arrest in the cell cycle and leading to inhibitory effect on GC proliferation (Shi et al., 2019).

SIRT1 can also assist with GC chemotherapy. In GC cells treated with oxaliplatin in xenograft tumor *mouse* model, SIRT1 may modulate chemotherapy resistance by influencing ferroptosis (Qu et al., 2023). Studies have found that in the *mice* model, GC tissues with high levels of SIRT1 expression exhibit increased sensitivity to cisplatin and 5-FU. Mechanistically, SIRT1 activates AMPK by deacetylating LKB1. AMPK promotes the nuclear translocation of FOXO3 and enhances its transcriptional activity. In turn, FOXO3 increases the expression and activity of AMPKα by directly binding to its promoter and activating transcription. This coordinated effect further inhibits chemoresistance and cancer stem cell (CSC) properties (Qu et al., 2023; An et al., 2020). Meanwhile, upregulated SIRT1 can increase the inhibition of CREB(cAMP Response Element-Binding Protein), reduce its downstream product ABCG2, a protein associated with drug efflux and resistance, and ultimately increase the sensitivity of Lgr5+GCSCs to cisplatin in GC cells (Zhang L. et al., 2017). Regarding CREB, a study has shown that SIRT1 can inhibit the expression of CA9 (carbonic anhydrase IX) by forming a complex with the adapter protein p300 and CREB. This interaction leads to a decrease in both the mRNA and protein levels of CA9, thereby suppressing the growth of GC in *nude mice* model (Wang et al., 2015). Additionally, in mouse models deficient in SETD2—a histone methyltransferase that catalyzes H3K36 trimethylation—tumorigenesis is promoted due to the inhibition of the SIRT1/FOXO signaling pathway, highlighting SIRT1’s role downstream of SETD2 in regulating tumor progression (Feng et al., 2023).

Beyond the pathways previously discussed, SIRT1 is also implicated in the STAT3/MMP-13 signaling pathway. In *mice* models also showed depleted of SIRT1, the expression levels of phosphorylated STAT3, acetylated STAT3, and(matrix metalloproteinase 13)MMP-13 are significantly upregulated. Activation of the STAT3/MMP-13 signaling pathway promotes the progression of GC (Zhang et al., 2019). Furthermore, NF-κB, which is extensively studied in CRC, is also regulated by SIRT1 in GC. SIRT1 induces G1 phase arrest through the NF-κB/Cyclin D1 signaling pathway, thereby inhibiting tumor proliferation (Yang et al., 2013b). Additionally, analysis of clinical samples has shown that SIRT1 directly binds to and deacetylates the transcription factor c-JUN, thereby inhibiting its transcriptional activity, which in turn reduces tumor size, inhibits tumor infiltration, and decreases the likelihood of lymph node metastasis and clinical progression in GC (Dong et al., 2018).

**FIGURE 3:**
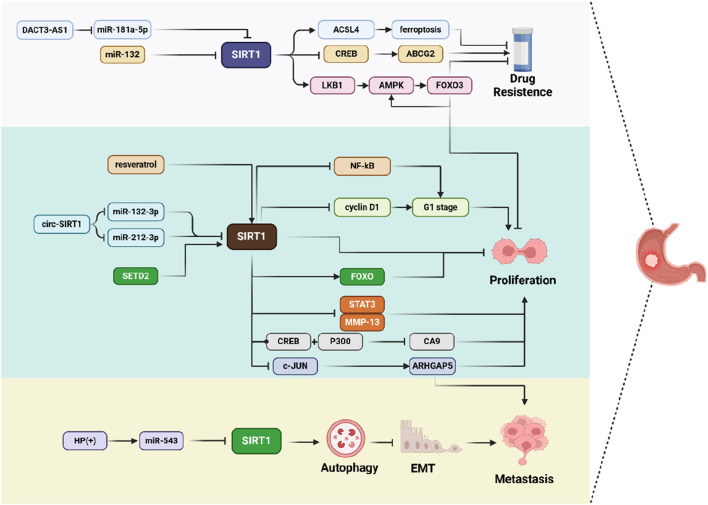
Schematic representation of SIRT1 inhibits GC progression in multiple ways. SIRT1 is involved in various molecular pathways, such as ferroptosis, cell cycle regulation, autophagy, and EMT, thereby influencing cell drug resistance, proliferation, and metastasis.

In contrast to the tumor-promoting effects of SIRT1 in GC, the mechanisms by which SIRT1 exerts its inhibitory effects on GC are remarkably diverse. Whether it is through the suppression of tumor growth and metastasis, or by playing a pivotal role in overcoming chemotherapy resistance, SIRT1 clearly demonstrates its anti-cancer potential in GC (Figure 3).

## 5 SIRT1 and CRC

In CRC, SIRT1 remains an important research hotspot. Despite some studies indicating that SIRT1 expression is downregulated, the majority have shown that SIRT1 is upregulated and deemed vital for all stages of CRC tumorigenesis. We will also discuss the role of SIRT1 in CRC from both positive and negative perspectives.

### 5.1 SIRT1 promotes CRC progression in multiple ways

In GC, numerous studies have confirmed that SIRT1 can serve as a prognostic molecular marker to predict patient outcomes, and similar studies have also been conducted in CRC. There are many clinical statistical researches demonstrate that overexpression of SIRT1 was significantly positively correlated with poor prognosis and advanced stage of CRC (Lv et al., 2014; Lee et al., 2021; Jiang et al., 2014). Interestingly, one study reported that this association is less common in young patients (Lee et al., 2021). What’s more, SIRT1 genotypes are also associated with CRC subtypes, especially (Microsatellite Instability)MSI-high phenotype CRC (Nosho et al., 2009; Hrzic et al., 2020). Further studies have explored the reasons behind these phenomena through various molecular mechanisms.

#### 5.1.1 SIRT1 deacetylates p53 and inhibits its activity

P53 is one of the important tumor suppressor proteins for CRC, and its activity can be regulated by SIRT1 via deacetylation. Knocking out the SIRT1 gene can increase the expression level of P53 in CRC cell (Chen et al., 2014; Stunkel et al., 2007; Vaziri et al., 2001). NAMPT is a significant oncogene that targets P53 via SIRT1. c-MYC, in conjunction with NAMPT, promotes the production of the SIRT1 cofactor NAD^+^. Notably, c-MYC can bind to the SIRT1 inhibitory protein DCB1. Simultaneously, SIRT1 can inhibit c-MYC induced cell apoptosis mediated by p53 (Menssen, 2013; Pan et al., 2019). Besides, long non-coding RNAs HNF1A-AS1 and H19 can regulate the expression of miRNA-34a and miR-194-5p by functioning as competing endogenous RNAs (ceRNAs). These miRNAs then directly target the SIRT1/P53 signaling axis, leading to the inhibition of p53 activity in both *nude mice* models and cells (Fang et al., 2017; Wang et al., 2018). Additionally, P53 requires modification to exert its proapoptotic function, specifically the acetylation of Lys382. Nox1 can upregulate the expression of SIRT1, and SIRT1 targets P53 Lys382 and mediates its deacetylation to suppress p53 proapoptotic transcriptional activity (Puca et al., 2010). These oncogenes contribute to the anti-apoptotic activity of CRC cells, highlighting the complex and critical role of SIRT1-mediated deacetylation of p53 in tumor suppression and proliferation in CRC.

SIRT1/P53 signaling axis is also one of the hotspots in CRC treatment. Numerous therapeutic mechanisms targeting SIRT1 have been explored. SIRT1 inhibitors, such as 4bb (Ghosh et al., 2017), MHY2245 (Kang et al., 2022a), MHY2251 (Kang et al., 2022b), directly target SIRT1 to produce a series of cytotoxic effects in *HCT116* cells. These studies have shown that CRC cells undergo apoptosis through three signaling pathways after treatment with SIRT inhibitors: the SIRT1/P53 pathway via deacetylation (Ghosh et al., 2017), the c-Jun N-terminal kinase (JNK) pathway and its downstream regulated caspases (Kang et al., 2022b), and the Fas/FasL pathway (Kang et al., 2022b). Differently, MHY2245 is closely bound up with CRC cell cycle by regulating cyclin B1, cell division cycle protein 2 (Cdc2), and Cdc25c. Another study injected *HCT116* cells into *nonobese diabetic/severe combined immunodeficiency (NOD/SCID) mice* and indicated that the antipsychotic drug chlorpromazine (CPZ) has potential therapeutic effects on CRC through its interaction with SIRT1. CPZ directly induced SIRT1 ubiquitination and promoted SIRT1 protein degradation, then increase the acetylation level of P53 and lead to apoptosis of CRC cells (Lee et al., 2015).

**FIGURE 4:**
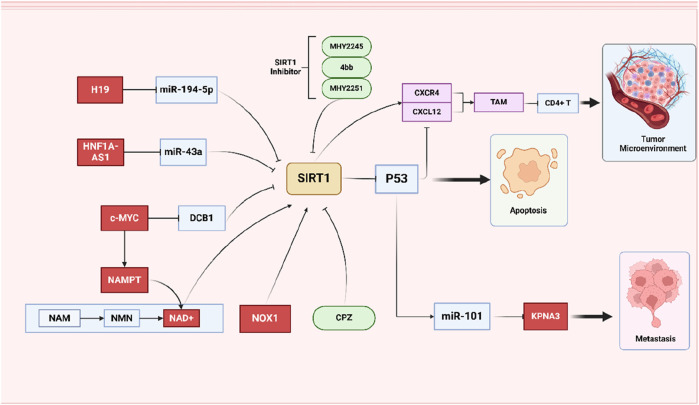
Schematic representation of the role of SIRT1/P53 signing pathways on CRC. SIRT1/P53 axis interacts with various molecules and pathways to influence CRC cell behavior. Simultaneously emphasizing the role of SIRT1 inhibitors and the regulation of the tumor microenvironment by SIRT1.

On the whole, it appears that the SIRT1/P53 axis primarily influences CRC through the mediation of cell apoptosis. However, recent studies have also highlighted the role of p53 in the immune microenvironment and metastasis of tumor cells. In CRC xenograft tumors established in nude mice, overexpression of SIRT1 leads to the deacetylation of P53, increases the expression of CXCL12, and enhance CXCR4 expression in the tumor microenvironment. Subsequently, SIRT1 regulates the migration of tumor-associated macrophages (TAMs) through the CXCR4/CXCL12 pathway, which inhibits the proliferation and activity of CD8^+^ T cells (Fang et al., 2022). Another study reported that in nude mice, the deacetylation of P53 results in the downregulation of miR-101, thereby weakening the inhibitory effect on its target gene KPNA3. This mechanism allows SIRT1 to promote tumor cell migration, invasion, and EMT via the p53/miR-101/KPNA3 pathway (Wang et al., 2023) (Figure 4).

#### 5.1.2 SIRT1 activates the Wnt/β-catenin signaling pathway to promote the progression of CRC

The Wnt signaling pathway, particularly the canonical Wnt/β-catenin pathway, is a critical regulator of various cellular processes, including cell migration, apoptosis, and proliferation (Cheng et al., 2019). The canonical Wnt/β-catenin signaling pathway is implicated in the onset of CRC and is considered a promising therapeutic target (Zhao H. et al., 2022) Abnormal activation of the Wnt/β-catenin signaling pathway can promote the progression of CRC (Sun et al., 2024). Overexpressed SIRT1 directly activates Wnt/β-catenin signaling pathway and promotes the occurrence of CRC (Zhang et al., 2023). As the oncogenes of CRC, both nuclear enriched abundant transcript 1 (NEAT1) and Pseudouridine synthase 7 (PUS7) are involved in the initiation of the tumor through SIRT1/Wnt/β-catenin axis in the *nude mice* model. NEAT1 is a Long non-coding RNA which can competitively bind to miR-34a and upregulate the expression of SIRT1. PUS7 can be combined with SIRT1 to stabilize it and enhance its functionality (Zhang et al., 2023; Luo et al., 2019). Besides, downstream gene c-MYC of Wnt/β-catenin signaling pathway has been reported to be closely related to malignant transformation in the serrated route to CRC (Menssen, 2013; Kriegl et al., 2012; Brandl et al., 2018; Kriegl, 2013). After extensive analysis of CRC patient tissues and cell lines, researchers found high expression levels of c-MYC and SIRT1 can be observed in most serrated lesions, the majority of which are related to KRAS and BRAF mutations. However, serrated lesions without these two gene mutations are believed to be induced by the Wnt/β-catenin signaling pathway. Once activated, the Wnt/β-catenin signaling pathway leads to an increase in nuclear β-catenin, which induces c-MYC transcription. This process can enhance SIRT1 activity through a feedback loop involving c-MYC, NAMPT, and SIRT1 (Kriegl et al., 2012; Brandl et al., 2018). In conclusion, Wnt/β-catenin/c-MYC/NAMPT/SIRT1 axis crucially contribute to onset and development of serrated route to CRC. In addition to the occurrence of cancer, SIRT1/Wnt/β-catenin signaling pathway is also involved in many other stages of CRC. For metastasis, HNF1A-AS1 inhibits miR-34a/SIRT1/p53 feedback loop and activates Wnt/β-catenin signaling pathway to promote CRC cell EMT and metastasis (Fang et al., 2017). For CRC cell metabolism, SIRT1 expression is upregulated in a glucose-deficient tumor microenvironment, leading to the deacetylation of β-catenin. This modification facilitates the translocation of β-catenin from the nucleus to the cytoplasm. Consequently, glycolysis is weakened, and fatty acid oxidation (FAO) is activated to provide an alternative energy supply. This metabolic shift has been observed both in cell cultures and in nude mice xenograft models (Wei et al., 2023). For chemotherapy, the anticancer effect of sodium salt of butrin (a novel compound isolated from Butea monosperma flowers) was found to be achieved by downregulating SIRT1 to inhibit Wnt/β-catenin signaling pathway activity in *SW480* cells (Subramaniyan et al., 2017). In summary, from these studies, as one of the targets of SIRT1, Wnt/β-catenin signaling pathway also plays an assignable role in the carcinogenesis and development of CRC.

#### 5.1.3 SIRT1 mediated EMT of CRC

Remote metastasis of CRC is a major cause of patient mortality. Approximately 20% of patients present with distant metastasis at initial diagnosis, and 50%–60% of those with primary CRC will eventually develop metastasis, commonly to the liver, lungs, and bones (Benson et al., 2017; Li et al., 2017). EMT, a process where epithelial cells transition to a mesenchymal phenotype, is crucial for metastasis. This transition increases cell mobility, invasiveness, and anti-apoptotic properties by disrupting tight junctions between tumor cells (Shin et al., 2023; Lu et al., 2023). SIRT1 is a key regulator in EMT. In tumor-bearing *mice*, Lipopolysaccharide-induced tumor necrosis factor alpha factor (LITAF) acts as a tumor suppressor gene, promoting FOXO-1 expression to inhibit SIRT1, thereby upregulating epithelial marker E-cadherin and downregulating mesenchymal marker N-cadherin. Conversely, silencing LITAF leads to high SIRT1 expression, promoting EMT (Guan et al., 2023). Additionally, NAMPT, an oncogene associated with CRC, induces EMT-related genes (TWIST1, VIM, SNAI1) and enhances SIRT1 activity via the NAD salvage pathway. However, inhibiting SIRT1 can reverse this effect in cell lines and nude *mice* (Lucena-Cacace et al., 2018). Meanwhile, Ubiquitin-conjugating enzyme E2 variant 1 (UBE2V1) degrades SIRT1 through ubiquitination, reducing its promotion of autophagy and inducing EMT via an autophagy-related mechanism in orthotropic *mouse* xenograft model of lung metastasis (Shen et al., 2018).

As a pivotal signaling hub, SIRT1 receives signals from gene transmission and activates multiple molecular downwards, such as Wnt/Fos-related antigen 1 (Diesch et al., 2014; Cheng et al., 2016), P53/ miR-101/KPNA3 (Wang et al., 2023), and NF-κb (Pyo and Kim, 2019). In *nude mice* xenograft model and cells, Fra-1, a downstream molecule of the Wnt signaling pathway, is considered the gatekeeper of EMT in CRC cell metastasis. As previously discussed, SIRT1 activates the Wnt signaling pathway to upregulate the expression of Fra-1, ultimately inducing the EMT process in tumors (Diesch et al., 2014; Cheng et al., 2016). What’s more, researchers have constructed a xenograft model of CRC and a metastasis model using *nude mice* and found that SIRT1 can also deacetylate P53 to downregulate the expression of miR-101, thereby upregulating the expression of its target gene KPNA3. The results of western blot analysis indicated that overexpression of KPNA3 can increase E-cadherin and occluding levels, while reducing vimentin, N-cadherin, and fibronectin levels (Wang et al., 2023). Regarding NF-κb, there is a study counted 261 CRC tissue samples, analyzed the relationship between NF-κb and CRC by immunohistochemical method, and found that the expression of NF-κb was significantly correlated with the expression of SIRT1 and SNAIL, a marker of EMT (Pyo and Kim, 2019). Interestingly, except SIRT1, its pre-mRNA circ-SIRT1 is also related to CRC metastatic. *Wang et al* analyzed *HCT116* and *HT29* cell lines and reported that circ-SIRT1 knockdown inhibited the expression of N-cadherin and vimentin. Mechanically, they found circ-SIRT1 could bind to eukaryotic tTranslation initiation factor 4A3 (EIF4A3), which plays a role in mRNA quality control before translation initiation. Consequently, circ-SIRT1 suppressed the recruitment of EMT-related protein mRNAs by EIF4A3, thereby further regulating the progression of EMT (Wang X. et al., 2021).

#### 5.1.4 Non coding RNA affects the progression of CRC through SIRT1

According to the latest research, 75% of the human genome can be transcribed into RNA, while only 3% can be translated into proteins, the RNA that is not translated into proteins is referred to as non-coding RNA(ncRNA) (Kimura, 2020). ncRNAs can be divided into microRNA (miRNA) and long ncRNA (lncRNA) based on their length, both of them are closely related to malignant tumor (Yan and Bu, 2021; Ma Y. et al., 2018; Kopp and Mendell, 2018). There is abundant evidence to confirm that miRNAs are involved in the occurrence (Wang et al., 2020), proliferation (Wang et al., 2023), invasion (Luo et al., 2019), resistance (Wang et al., 2023; Wang et al., 2018; Wu et al., 2019; Zhao B. et al., 2022), and metastasis of CRC (Sun et al., 2017; Fang et al., 2017; Shen et al., 2016) through different mechanisms. Of course, the assistance of SIRT1 is indispensable. It is worth mentioning that lncRNAs and miRNAs sometimes antagonize each other and fulfill their respective roles (Fang et al., 2017).

In both cell and mouse models, researchers have reported a consistent mechanism involving miRNAs functioning as competing endogenous RNAs (ceRNAs) to sponge other non-coding RNAs (ncRNAs). Examples include miR-34a/ HNF1A-AS1 (Fang et al., 2017), miR-34a/NEAT1 (Luo et al., 2019), miR-34a/GAS5 (Zhang et al., 2021), miR‐135a‐5p/FOXD3‐AS1 (Wu et al., 2019), miR-194–5p/H19 (Wang et al., 2018). All of these miRNAs were negatively correlated with the expression of SIRT1. Subsequently, lncRNAs, acting as ceRNAs for these miRNAs, functioned as oncogenes. They upregulated the expression of SIRT1 by sponging miRNAs and further participated in the processes of CRC. As a result of SIRT1 overexpression, different pathways, such as Wnt (Luo et al., 2019) and P53 (Fang et al., 2017) are activated and transmit signals to the downstream. Additionally, some lncRNAs directly exert their effects through SIRT1. For example, H19 mediates autophagy via SIRT1 (Wang et al., 2018). Based on the analysis of CRC tissues and corresponding hepatic metastasis tissues, the downregulation of miR-199b is closely related to the distal metastasis and high TNM stage of CRC. miR-199b can downregulate the expression of SIRT1, further enhance the acetylation of transcription factor CREB. Then the activated CREB acts on the promoter of KISS1, thereby upregulating the expression of KISS1, a metastasis suppressor (Shen et al., 2016). Similarly, low expression of miR-138 also symbolizes poor prognosis in CRC patients, as miR-138 can also target the 3′UTR of SIRT1 gene and inhibit its expression (Kang et al., 2021). MiRNA-34a can also downregulate the expression of SIRT1, exerting anti-cancer effects. Researchers evaluated in the *rat* model of azoxymethane (AOM)-induced CRC and found that is alsothe mechanism by which Catalpol can treat CRC (Qiao et al., 2020).

Some miRNAs are regulated by SIRT1, serving as a link in the downstream signaling of SIRT1. SIRT1 can deacetylate the promoter of miR-1185-1 and inhibit its expression, which targets CD24 gene 3′UTR to suppress its expression. In this way, SIRT1 can increase stemness in CRC by promoting CD24 expression in the mouse xenograft model (Wang et al., 2020). Likewise, SIRT1 deacetylates P53, thereby reducing the expression of miR-101, relieving miR-101's inhibition of the target gene KPNA3 and promoting the progression of CRC (Wang et al., 2023). In addition, other studies have also reported that SIRT1 can participate in tumor immune escape (Meng et al., 2021), drug resistance (Zhao B. et al., 2022), and inhibition of CRC through miRNA (Sun et al., 2017). From these various studies, we can learn that ncRNA, especially miRNA, plays an important role in various stages of CRC together with SIRT1 (Table 1).

**TABLE 1:** The effect of miRNA on CRC through SIRT1. miRNAs and lncRNAs regulate SIRT1 expression by acting as ceRNAs or by directly targeting SIRT1, thereby impacting CRC progression, metastasis, and resistance.

ncRNA	Mechanisms	Outcomes	Refence
miR-34a/HNF1A-AS1	HNF1A-AS1 as ceRNA sponge miR-34a, promoting upregulation of SIRT1 expression	Promote the metastasis of CRC	Fang et al. (2017)
miR-34a/NEAT1	NEAT1 as ceRNA sponge miR-34a, promoting upregulation of SIRT1 expression	Promote the viability and invasion of CRC	Luo et al. (2019)
miR-34a/GAS5	GAS5 as ceRNA sponge miR-34a, promoting upregulation of SIRT1 expression	Promote the autophagy of CRC	Zhang et al. (2021)
miR-135a-5p/FOXD3-AS1	FOXD3-AS1 as ceRNA sponge miR-135a-5p, promoting upregulation of SIRT1 expression	Promote the resistance and autophagy of CRC	Wu et al. (2019)
miR-194-5p/H19	H19 as ceRNA sponge miR-194-5p, promoting upregulation of SIRT1 expression	Promote the tumor progression of CRC	Wang et al. (2018)
miR-199b	miR-199b directly downregulates SIRT1 expression	Inhibit the metastasis of CRC	Shen et al. (2016)
miR-138	miR-138 directly downregulates SIRT1 expression	Inhibit the tumor progression of CRC	Kang et al. (2021)
miR-34a	miR-34a directly downregulates SIRT1 expression	Inhibit the immune evasion of CRC	Qiao et al. (2020)
miR-1185-1	SIRT1 inhibits miR-1185-1 and upregulates CD24	Promote stemness in CRC	Wang et al. (2020)
miR-101	SIRT1 inhibits miR-101 and upregulates KPNA3	Promote resistance, growth, metastasis of CRC	Wang et al. (2023)
miR-15b-5p	SIRT1 inhibits miR-15b-5p and upregulates ACOX1	Inhibit the metastasis of CRC	Sun et al. (2017)
miR-20b‐3p	SIRT1 inhibits miR-20b‐3p and upregulates DEPDC1	Promote L-OHP resistance of CRC	Zhao B. et al. (2022)

#### 5.1.5 SIRT1 mediated chemotherapy resistance in CRC

In tumor treatment, single-target agents often induce drug resistance and reduce efficacy (Antolin et al., 2016). Combination drugs, which act via multiple pathways or targets, are the best solution (Benboubker et al., 2024). In CRC treatment, the FOLFOXIRI regimen (FOL: folinic acid, F: 5-FU, OX: oxaliplatin, IRI: irinotecan) is commonly used (Benson et al., 2017). 5-Fluorouracil (5-FU) is a cornerstone in CRC treatment, primarily inhibiting thymidylate synthase (TS) to disrupt DNA synthesis by depleting intracellular deoxynucleotide pools (Vodenkova et al., 2020). Oxaliplatin, a platinum-based drug, exerts anti-CRC effects by forming active platinum complexes that bind to DNA, causing intra- and inter-strand crosslinks, leading to DNA damage and apoptosis (Zhang C. et al., 2022). As classic chemotherapy drugs for CRC, both oxaliplatin and 5-FU are closely related to SIRT1 in terms of resistance.

Regarding oxaliplatin, SIRT1 influences the resistance of CRC cells through multiple molecules. Some studies conducted CRC cell and *nude mice* model and identified the potential mechanism of the oxaliplatin-resistant (OR) (Zhao B. et al., 2022; Shen et al., 2016; Fang et al., 2018). In those studies, researchers stated that SIRT1 was increased in the OR-CRC tissues and the knockout of SIRT1 significantly enhanced the sensitivity of CRC tissues to oxaliplatin. In OR-CRC tissues, the aberrantly expressed SIRT1 protein binds to the promoter of miR-20b-3p and inhibits its transcription. This action relieves the repression of DEPDC1 expression, thereby increasing the cellular level of DEPDC1 and enhancing resistance to oxaliplatin (Zhao B. et al., 2022; Shen et al., 2016). It was also found that the expression levels of nuclear transcription factor Y subunit β(NFYB) and E2F transcription factor 1 (E2F1) in OR-CRC cells were significantly higher than those in non-resistant cells. High expression of NFYB can activate the transcription of E2F1 gene and E2F1 mediates oxaliplatin resistance by promoting the expression of Checkpoint Kinase 1 (CHK1). In the NFYB/ E2F1/CHK1 axis, SIRT1 can deacetylate E2F1 to inhibit its pro apoptotic activity, which is essential for maintaining oxaliplatin resistance in OR-CRC cells (Fang et al., 2018). Nucleotide excision repair (NER) pathway also plays an important role in the mechanism of oxaliplatin resistance. Studies have shown that the deacetylation of xeroderma pigmentosum complex group A (XPA) by SIRT1 can promote the NER pathway (Fan and Luo, 2010). In addition, Hypermethylated in Cancer 1 (HIC1) is a sequence specific transcriptional repressor that directly binds to the SIRT1 promoter and inhibits SIRT1 transcription. Consequently, the mutational inactivation of HIC1 results in the upregulation of SIRT1 expression and the activation of the NER pathway, as confirmed in a cohort study (Okazaki et al., 2017).

Research on 5-FU resistance is inseparable from ncRNA, lncRNA H19 increases 5-FU resistance through SIRT1 mediated autophagy in CRC cells (Wang et al., 2018). At the same time, SIRT1 can also reduce p53 through deacetylation, and then downregulate the expression of miR-101, and increase the expression of miR-101 target gene KPNA3, to enhance the resistance to 5-FU (Wang et al., 2023). Interestingly, a study found that the high expression of miR-653-3p was related to the drug resistance of 5-FU in the xenograft *mice* model. Mechanically, miR-653-3p inhibited the expression of SIRT1 by directly interacting with the 3′UTR of SIRT1. Subsequently, miR-653-3p promoted the phosphorylation of STAT3 and the expression of Twist1, a transcription factor that can promote chromosomal instability, then inhibited apoptosis (W et al., 2023).

The alteration of metabolic pathways in CRC cells is also one of the mechanisms underlying SIRT1-induced drug resistance. PGC1-α is a key regulatory factor for mitochondrial function and biosynthesis. Following chemotherapy, researchers have observed an increase in mitochondrial number and oxygen consumption in CRC cells, along with upregulation of SIRT1 expression. SIRT1 activates PGC1 α through deacetylation, thereby shifting the cellular metabolic pathway from glycolysis to oxidative phosphorylation. This metabolic reprogramming enhances the tumor’s resistance to chemotherapy, as demonstrated in models constructed using *CBy.Cg-Foxn1nu/J* mice or *NOD/SCID* mice (Vellinga et al., 2015; Witherspoon et al., 2019). Consistent findings have been reported in studies analyzing the genomes of hundreds of CRC tissue samples (Vellinga et al., 2014). Additionally, some anti-cancer agents, such as Manuka honey and Xanthohumol, exert their effects by inhibiting the SIRT1/PGC1-α axis (Sastre-Serra et al., 2019; Afrin et al., 2018). Moreover, upon attack by anti-cancer therapies, SIRT1 deacetylates β-catenin, promoting its translocation from the nucleus to the cytoplasm. This process weakens glycolysis and is positively correlated with fatty acid oxidation (Wei et al., 2023). Conversely, conflicting results have been reported in the literature. In a *nude mice* model, FOXQ1, which is highly expressed in CRC, acts on the SIRT1/β-catenin axis, causing β-catenin to transfer from the cytoplasm to the nucleus. This nuclear accumulation of β-catenin results in radiation resistance of tumor cells (Yang et al., 2022).

**FIGURE 5:**
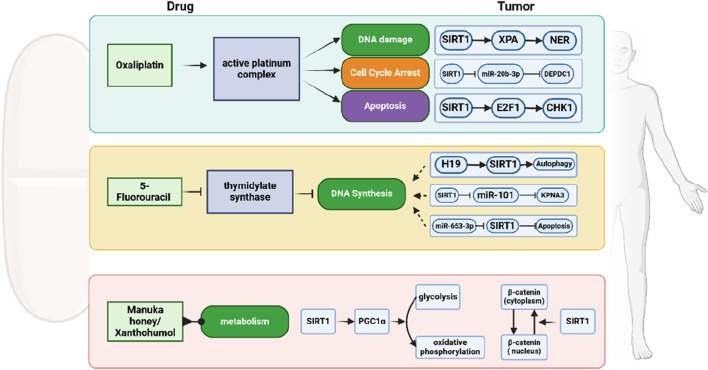
Schematic representation of chemotherapy resistance of CRC mediated by SIRT1. SIRT1 modulates the effects of chemotherapy drugs like Oxaliplatin and 5-Fluorouracil, as well as natural compounds such as Manuka honey and Xanthohumol, by affecting pathways including DNA damage, cell cycle arrest, apoptosis, and autophagy.

Other studies have reported that the combination of a SIRT1 inhibitor with anti-CRC drugs such as 5-FU, SN-38, or oxaliplatin can promote cell apoptosis and enhance the anti-tumor effect (Ueno et al., 2013). For example, SIRT1 inhibitors sensitize TP53 mutant (MUT) *SW620* cells to a variety of chemotherapeutic drugs (Yang et al., 2020). However, different scenarios have been reported in another CRC cell line. In *HCT116* cells harboring wild-type TP53, SIRT inhibitors showed antagonistic effects with a variety of chemotherapeutic drugs, including cisplatin, 5-fluorouracil, oxaliplatin, gefitinib, LY294002, and metformin, thereby reducing the antitumor effects of these drugs. These findings highlight the complex role of SIRT1 in CRC treatment and suggest that the efficacy of SIRT1 inhibitors may depend on the TP53 status of the cancer cells (Figure 5).

### 5.2 SIRT1 inhibits CRC progression in multiple ways

Although many studies have provided substantial evidence that SIRT1 plays a positive role in the occurrence and development of tumors, some studies have reached the opposite conclusion. For example, while high levels of SIRT1 are often associated with advanced-stage tumors, as many studies have shown, the opposite has also been observed. High levels of SIRT1 expression are found in normal colonic mucosal tissues and benign adenomas (Kabra et al., 2009), whereas a reduction in SIRT1 expression is seen in adenocarcinoma, metastatic tissues, and advanced stage Ⅳ tumors (Jang et al., 2012). This suggests that SIRT1 may exert tumor-suppressive effects during certain stages of cancer development.

In the research on SIRT1’s role in inhibiting cancer, two molecules, β-catenin and NF-κB, are particularly crucial. The anti-cancer effects of caloric restriction (CR) in mammals have been well-documented (Lien et al., 2021), with SIRT1 playing a significant role in energy metabolism (Mich et al., 2007). Concurrently, CR is associated with an upregulation of SIRT1 expression within the intestinal tract. Furthermore, SIRT1 inhibits its ability to activate transcription and drive cell proliferation by deacetylating β-catenin, and promotes the cytoplasmic localization of β-catenin. In 81 human colon tumor specimens and *APC* ^*min/+*^ *mouse* model, the presence of SIRT1 was significantly negatively correlated with the oncogenic form of β-catenin (Firestein et al., 2008). DBC1 can promote CRC development by facilitating the formation of the LEF1-β-catenin complex and the transcription of PROX1, a transcription factor induced by the Wnt/β-catenin pathway that can drive colorectal malignant transformation. These effects are achieved through the inhibition of SIRT1 by DBC1, which has been examined in the *nude mice* model (Yu et al., 2016). Moreover, this mechanism has also been confirmed in drug development (Harada et al., 2016). NF-κB is an important transcription factor that plays a central role in the regulation of immune response and inflammatory process. Its abnormal activation can promote cell proliferation, inhibit apoptosis, and promote EMT to support tumor cell growth and invasion in the *Apc* ^*Min*^ *mouse* model (Porta et al., 2018). This is why NF-κB is a target for many anti-tumor drugs, which generally work by increasing the expression and activity of SIRT1 (El-Kott et al., 2021; Zhang et al., 2014; Zhou et al., 2019; Buhrmann et al., 2016). The upregulated SIRT1 can directly deacetylate NF-κB and inhibit its activity, ultimately achieving the purpose of anti-tumor.

There are also studies where the tumor suppressor mechanism of SIRT1 is more complex. sad1/unc84 domain protein-2 (SUN2) is a key component connecting the nucleoskeleton and the cytoskeleton complex, and it may be associated with metastasis in various cancers (Liu et al., 2021). In CRC, high expression of SUN2 is linked to a favorable prognosis. This is attributed to the regulation of the acetylation state of methyl CpG binding protein 2 (MeCP2) by a complex composed of SUN2 and SIRT1. This regulation increases MeCP2’s binding activity to the brain-derived neurotrophic factor (BDNF) promoter, thereby reducing BDNF expression. The decreased activity of bdnf/ tropomyosin-related kinase B signaling pathway ultimately reduces the metastatic ability of cells in *nude mice* model (Liu et al., 2021). SIRT1 can also participate in the process of suppressing cancer by regulating downstream molecules. In the analogous xenograft model constructed in *nude mice*, SIRT1 weakens the transcriptional activation of the miR-15b-5p promoter by deacetylating the AP-1 complex. The downregulated miR-15b-5p cannot exert an inhibitory effect on its target, acyl-CoA oxidase 1 (ACOX1). High-levels ACOX1 can inhibit the proliferation and metastasis of CRC cells (Sun et al., 2017). In addition, studies have reported that SIRT1 is involved in the anti-tumor mechanisms of vitamin D. Calcitriol, an active metabolite of vitamin D, can promote the activation of SIRT1 through auto-deacetylation, thereby enhancing SIRT1’s deacetylation activity (García-Martínez et al., 2023).

These studies collectively highlight the anti-tumor effects of SIRT1, which also suggested that SIRT1 has a double-sided role in the occurrence and development of CRC.

## 6 Conclusion

**FIGURE 6:**
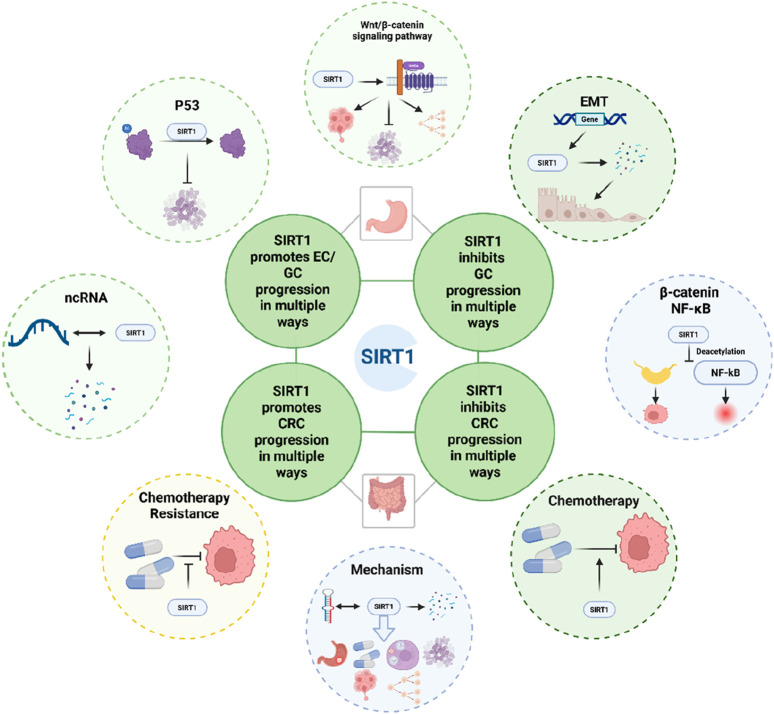
Schematic representation of the role of SIRT1 in the development of gastrointestinal tumors, provides a concise summary of the key molecules and cellular processes influenced by SIRT1 in the context of CRC and GC.

According to epidemiological statistics, gastrointestinal tumors rank high in both incidence and mortality rates. Since they typically present with significant clinical symptoms only in the advanced stages, they are characterized by late detection and poor treatment outcomes. Therefore, it is essential to identify highly sensitive and specific biomarkers for early diagnosis. This review centers on SIRT1’s complex role in gastrointestinal tumors, especially esophageal cancer (EC), gastric cancer (GC), and colorectal cancer (CRC), highlighting its dual potential as both a tumor promoter and suppressor. Understanding SIRT1’s intricate mechanisms is crucial for its context-specific function (Figure 6).

In EC, SIRT1 consistently drives the progression of tumor. In GC, SIRT1’s role is not straightforward. Like CRC, SIRT1 can also regulate similar molecules that affect tumor progression in GC cells. However, we have noticed that there are more studies on the anticancer effect of SIRT1 in GC, which may be related to SIRT1 mediated ferroptosis and *Helicobacter pylori* infection. Further research is needed to reach specific conclusions. Similarly, in CRC, SIRT1 is involved in tumor development, proliferation, invasion, and metastasis through important molecules such as P53, Wnt/β-catenin signaling pathway, and ncRNA. We also found that SIRT1 plays a crucial role in the progression of tumor EMT and drug resistance. However, contrasting findings suggest that SIRT1 can also act as a tumor suppressor by inhibiting β-catenin and NF-κB signaling pathways. This duality underscores the complexity of SIRT1’s function and suggests that its role may vary depending on the stage of cancer and the specific molecular context.

Future research should focus on elucidating the specific molecular contexts in which SIRT1 exerts its pro- or anti-tumorigenic effects. This includes investigating the role of SIRT1 in different stages of cancer development and identifying biomarkers that can predict SIRT1’s function in individual patients. Moreover, the development of selective SIRT1 modulators is also an important area of research. Currently, there are many SIRT1-related drugs, such as SIRT1 inhibitors. However, specific clinical trials in this area remain largely unexplored.

In conclusion, the dual roles of SIRT1 in gastrointestinal tumors present both challenges and opportunities for cancer research and therapy. By unraveling the complex mechanisms through which SIRT1 operates, we can pave the way for more effective and personalized treatments for gastrointestinal tumors.
